# Comparison of Laser Iridotomy and Lensectomy Outcomes for Acute Primary Angle Closure

**DOI:** 10.1155/2022/6959479

**Published:** 2022-05-30

**Authors:** Kei Iijima, Kazutaka Kamiya, Yoshihiko Iida, Nobuyuki Shoji

**Affiliations:** ^1^Department of Ophthalmology, School of Medicine, Kitasato University, Tokyo, Japan; ^2^Visual Physiology, School of Allied Health Sciences, Kitasato University, Tokyo, Japan

## Abstract

**Purpose:**

To compare the clinical outcomes of the different treatments for acute primary angle closure (APAC).

**Methods:**

We retrospectively reviewed the clinical charts of 87 eyes of 87 patients undergoing treatment for APAC. We investigated the best spectacle-corrected visual acuity (BSCVA), intraocular pressure (IOP), corneal endothelial cell density (ECD), and secondary interventions after each treatment.

**Results:**

The pretreated IOP was 56.4 ± 9.0 mmHg. As the first treatment for APAC, all eyes underwent topical 2% pilocarpine and systemic mannitol administration. Subsequent laser iridotomy (LI) and lensectomy were necessary in 29 eyes (33%) and 35 eyes (40%), respectively. Bullous keratopathy developed in 1 eye (1%), and following glaucoma surgery was required in 7 eyes (8%). The BSCVA at the final follow-up was 0.16 ± 0.53 and 0.01 ± 0.20 logMAR (Mann–Whitney *U* test, *p*=0.149), the IOP was 12.8 ± 2.6, and 12.6 ± 2.9 mmHg (*p*=0.860), and the ECD was 2295.9 ± 658.2 and 2244.1 ± 622.0 cells/mm^2^ (*p*=0.735) in the LI and lensectomy groups, respectively.

**Conclusions:**

Approximately 26% of eyes with APAC were resolved after the initial medical treatment, and subsequent surgical treatments, such as LI and lensectomy, were required in 33% and 40% of eyes, respectively. We found no significant differences in the BSCVA, the IOP, or the ECD among LI and lensectomy treatment groups.

## 1. Introduction

Acute primary angle closure (APAC) has been widely acknowledged as one of the significant ophthalmic diseases requiring emergency treatment. As the initial treatment, patients developing APAC usually undergo topical pilocarpine and systemic mannitol administration. When the initial medical treatment does not dissolve the acute attack, the surgical interventions, including laser iridotomy (LI) or lensectomy, are necessary to resolve the pupillary block and subsequent intraocular pressure (IOP) rise. Individual ophthalmologists habitually select the emergency treatment option based on their skills and experiences with such treatments.

Until now, there have been several studies on the treatment outcomes of APAC [1–10]. However, as far as we can ascertain, detailed treatment outcomes for APAC have not been thoroughly compared according to each treatment option. Nevertheless, it may give us intrinsic insights for grasping the overall features and selecting the appropriate management for APAC in daily practice. The purpose of the current study is to retrospectively compare the treatment outcomes for APAC based on the received treatment in a cohort of post-APAC patients.

## 2. Materials and Methods

### 2.1. Study Population

The study protocol was registered with the University Hospital Medical Information Network Clinical Trial Registry (000048942). This retrospective study comprised a total of 87 eyes of 87 consecutive patients (17 men and 70 women; mean age ± standard deviation: 69.0 ± 9.0 years), who developed APAC between January 2006 and June 2020 at Kitasato University Hospital and who completed at least a 3-month follow-up. Eyes with any history of ocular surgery, ocular trauma, or other concomitant eye diseases except for glaucoma were excluded from the study. Only one eye was randomly chosen from each patient for statistical analysis when bilateral APAC occurred. This retrospective review of the clinical charts was approved by the Institutional Review Board at Kitasato University (B20-123) and followed the tenets of the Declaration of Helsinki. Our Institutional Review Board waived the requirement for informed consent for this retrospective study.

### 2.2. Treatment Protocol

As the initial treatment, all eyes underwent topical 2% pilocarpine and systemic mannitol administration. After that, we performed a surgical treatment, such as laser iridotomy (LI) or lensectomy, when APAC was not resolved after the first treatment. We mainly selected lensectomy when there were experienced surgeons in our institution and when the corneal edema was mild. Otherwise, we selected LI when there were no experienced surgeons or when the corneal edema was moderate to severe. In addition, we conducted prophylactic LI or lensectomy after that, even when the first treatment resolved APAC. For LI, laser iridotomy was performed in the superior peripheral region using a sequential argon laser and an Nd : YAG laser [11–14]. We applied the following settings for the argon laser preshot (power, 100 to 200 mW; spot size, 200 **μ**m; duration, 0.2 seconds) and the Nd : YAG laser (power, 2.0 to 5.0 mJ). For lensectomy, experienced surgeons performed standard phacoemulsification through a 2.8 mm temporal corneal incision. The surgical technique consisted of capsulorhexis, nucleus, and cortex extraction. If possible, we also performed subsequent nontoric intraocular lens (IOL) (PU-6, Kowa, Japan) implantation. The presence or absence of IOL implantation was determined by each surgeon based on the intactness of the capsular bag.

### 2.3. Outcomes Measures

We divided the subjects into two treatment groups (LI and lensectomy groups), based on the APAC treatment except for those who did not require the secondary surgical interventions after the initial treatment. We assessed the patient demographics, the selected treatment method, best spectacle-corrected visual acuity (BSCVA), intraocular pressure (IOP) measured with a Goldmann applanation tonometry, and corneal endothelial cell density (ECD) measured with a noncontact specular microscope (EM-3000, Tomey, Aichi, Japan) at the final follow-up after each treatment.

### 2.4. Statistical Analysis

We conducted statistical analyses using commercially available statistical software (Bell curve for Excel, Social Survey Research Information Co, Ltd., Tokyo, Japan). The Mann–Whitney *U* test was used to compare the BSCVA, the IOP, and the ECD of the two groups. The results are expressed as mean ± standard deviation, and a value of *p* < 0.05 was considered statistically significant.

## 3. Results

Table 1 shows the demographics of the study population before the treatment. The pretreated IOP was 56.4 ± 9.0 mmHg (95% confidence interval (CI), 38.7 to 74.1 mmHg). From the onset of subjective symptoms to the initial medical examination, the duration was 1.6 ± 2.7 days (95% CI, −3.7 to 6.9 days). The follow-up period was 15.5 ± 19.3 months (95% CI, −22.3 to 53.3 months).  Table 2 shows the prognosis of the BSCVA, the IOP, and the ECD in the whole study population. Of the 87 eyes developing APAC, 23 eyes (26%) were resolved after the initial treatment (topical 2% pilocarpine and systemic mannitol administration). The remaining 29 eyes (33%) and 35 eyes (40%) were treated by LI and lensectomy, respectively. In the whole study population, the mean BSCVA, the IOP, and the ECD at the final follow-up were 0.09 ± 0.43 logMAR, 12.6 ± 2.8 mmHg, and 2200.0 ± 644.7 cells/mm^2^, respectively. After lensectomy, IOL implantation was simultaneously performed in 17 of 35 eyes (49%); 15 eyes underwent in-the-bag fixation, and 2 eyes underwent out-of-the-bag fixation. IOL implantation was secondarily performed in 17 eyes of the remaining 18 eyes (4 eyes, in-the-bag fixation; 4 eyes, out-of-the-bag fixation; and 9 eyes, sulcus fixation), and only one eye was left as an aphakia, since this patient did not wish to undergo secondary IOL implantation due to optic nerve atrophy. We found zonular weakness during cataract surgery in 9 eyes but found no significant postoperative complications.

Bullous keratopathy developed in 1 eye (1%) after LI, and glaucoma surgery was required in 7 eyes (8%) (1 eye after nonsurgery, 5 eyes after LI, and 1 eye after lensectomy).  Table 3 shows the prognosis of the BSCVA, the IOP, and the ECD at the final follow-up, based on the received treatment. We found no significant differences in the BSCVA (Mann–Whitney *U* test, *p*=0.149), the IOP (*p*=0.860), or the ECD (*p*=0.735) between the two treatment groups.

## 4. Discussion

Our results showed no significant differences in visual acuity, IOP, or ECD among the two treatment groups in the present study.  Table 4 summarizes previous studies on the prognosis of the treatment for APAC. Jacobi et al. found that phacoemulsification with IOL implantation provided significantly better BSCVA and lower IOP than peripheral iridectomy for uncontrolled APAC [2]. Su et al. demonstrated, in a study of 16 eyes requiring lensectomy for APAC, that primary phacoemulsification with IOL implantation lowered the IOP, reduced the use of antiglaucoma medications, and improved the BSCVA in patients with APAC [4]. Park et al. stated that early lensectomy showed lower ECD loss than did LI during the 2 years, suggesting that it could serve as an excellent surgical option for APAC in terms of the ECD [5] Li et al. compared the outcomes of phacoemulsification and phacotrabeculectomy. They found that the improvement in BSCVA in the phacoemulsification group was significantly greater than that in the phacotrabeculectomy group, but that there were no significant differences in the IOP between them at the later follow-up (1 week thereafter) [8] Fea et al. reported morphological and functional outcomes of chronic angle closure in the eyes following APAC. They provided a comparison with their fellow eyes, showing that the IOP was 13.44 ± 2.78 and 13.89 ± 2.60 mmHg in angle closure and fellow eyes, respectively, and that 53% of fellow eyes developed chronic angle closure even when prophylactic LI was promptly performed [9] Noh et al. compared the outcomes of phacoemulsification alone and pars plana vitrectomy and phacoemulsification and found that combined surgeries are more effective and safer than phacoemulsification alone because of less operation time and fewer complications after APAC [10]. As far as we can ascertain, this is the first published study on the detailed prognosis of APAC treatments, according to each received treatment. Most previous studies are merely focused on the visual prognosis of lensectomy. Since the treatment option, the sample size, the follow-up period, the study design, and the outcome measures are individually different among these previous and current studies, we cannot directly compare the treatment outcomes of APAC, but visual outcomes in the present study were slightly better than, and the IOP and the ECD were almost equivalent to, those in previous studies, as shown in Table 3 [2, 4, 5, 8–10, 15] Lam et al. demonstrated that lensectomy was superior to LI in terms of reducing the IOP rise [1] Aung T et al. showed that the majority of eyes developing IOP rise after LI for APAC occurred within 6 months from the onset [16].

We assume that this information is simple but clinically meaningful for better understanding of the real-world prognosis of post-APAC patients in a clinical setting.

There are at least four limitations to this study. Firstly, we did not assess the ECD in all eyes since this study was performed in a retrospective fashion. Secondly, the minimum follow-up time was set at 3 months, and the observation period was 15.5 ± 19.3 months. A prospective study with a more extended observation period is still necessary to clarify this point. Thirdly, we did not confirm the exact etiology of APAC, including pupillary block, plateau iris, lens factor, and ciliary body factor, since we did not obtain images using anterior segment optical coherence tomography or ultrasound biomicroscopy in all eyes. Fourthly, the study was conducted in a retrospective, uncontrolled fashion. A prospective controlled study in a large cohort of another population would be ideal for confirming the authenticity of our results.

In summary, our results may support the view that approximately 30% of eyes with APAC were resolved after the initial medical treatment and that subsequent surgical therapies, such as LI and lensectomy, were necessary for 33% and 40% of eyes, respectively. In addition, there were no significant differences in the BSCVA, the IOP, or the ECD among the LI and lensectomy treatment groups in our case series. We believe that this information will be clinically helpful for understanding the overall prognosis of post-APAC patients.

## Figures and Tables

**Table 1:** Pretreated demographics of the study population for acute primary angle closure.

	Mean ± standard deviation (95% confidence interval)	Median
Age	69.5 ± 8.8 years (52.3 to 86.7 years)	70 years
Male : female	17 : 70	
Duration from the onset to the initial examination	1.6 ± 2.7 days (−3.7 to 6.9 days)	0.7 days
UCVA (logMAR)	1.45 ± 0.70 (0.08 to 2.83)	1.61
Intraocular pressure	56.4 ± 9.0 mmHg (38.7 to 74.1 mmHg)	56 mmHg
Axial length	22.67 ± 0.91 mm (20.88 to 24.46 mm)	22.55 mm
Emery–Little classification	Grade I : 7, II : 69, III : 10, IV : 1, V : 0	

UCVA = uncorrected visual acuity; logMAR = logarithm of the minimum angle of resolution.

**Table 2:** Posttreated outcomes of the best spectacle-corrected visual acuity, the intraocular pressure, and the endothelial cell density for acute primary angle closure in the whole study population.

	Mean ± standard deviation (95% confidence interval)	Median
Observation period	15.5 ± 19.3 months (−22.4 to 53.5 months)	6.2 months
BSCVA (logMAR)	0.09 ± 0.43 (−0.76 to 0.93)	−0.08
Intraocular pressure	12.6 ± 2.8 mmHg (7.1 to 18.0 mmHg)	12 mmHg
Endothelial cell density	2200.0 ± 644.7 cells/mm^2^ (936.3 to 3463.7 cells/mm^2^)	2371.0 cells/mm^2^
Complications	Bullous keratopathy in 1 eye, subsequent glaucoma surgery in 7 eyes	

BSCVA = best spectacle-corrected visual acuity; logMAR = logarithm of the minimum angle of resolution.

**Table 3:** Posttreated outcomes of the best spectacle-corrected visual acuity, the intraocular pressure, and the endothelial cell density for acute primary angle closure according to each received treatment.

Treatment group	LI	Lensectomy	*p* value
Number of eyes	29	35	
BSCVA (logMAR)	0.16 ± 0.53	0.01 ± 0.20	0.149
Intraocular pressure (mmHg)	12.8 ± 2.6	12.6 ± 2.9	0.860
Endothelial cell density (cells/mm^2^)	2295.9 ± 658.2	2244.1 ± 622.0	0.735

LI = laser iridotomy; BSCVA = best spectacle-corrected visual acuity; logMAR = logarithm of the minimum angle of resolution.

**Table 4:** Summary of previous studies on the treatment outcomes of acute primary angle closure.

Author	Treatment	Eyes	Follow-up	BSCVA (logMAR)	Intraocular pressure (mmHg)	Endothelial cell density (cells/mm^2^)
Jacobi et al. [2]	PI	32	≥6 months	0.55 ± 0.23	20.1 ± 4.2	N. A.
	Lensectomy	43		0.18 ± 0.21	17.8 ± 3.4	
Su et al. [4]	Lensectomy	16	3 months	0.73 ± 0.53	10.7 ± 2.8	
Park et al. [5]	LI	32	2 years	0.36 ± 0.24	15.7 ± 1.3	1880 ± 422
	Lensectomy	16		0.30 ± 0.29	13.2 ± 1.2	2113 ± 333
Li et al. [8]	Lensectomy	31	6 months	0.83 ± 0.21	13.7 ± 3.6	N. A.
Fea et al. [9]	LI or lensectomy	57	5 years	0.37 ± 0.49	13.4 ± 2.8	1923.9 ± 497.1
Noh et al. [10]	Lensectomy	16	6 months	0.23 ± 0.16	12.1 ± 2.2	1912.8 ± 457.7
	Vitrectomy and lensectomy	10		0.19 ± 0.14	13.3 ± 1.7	2317.7 ± 510.3
	LI	34		0.70 ± 0.75	15.0 ± 9.1	N. A.
Lin YH. et al. [15]	Lensectomy after LI	23	12 months	0.52 ± 0.41	13.0 ± 6.9	
	Lensectomy	24		0.57 ± 0.43	12.7 ± 17.9	
Current	LI	29	≥3 months	0.16 ± 0.53	12.3 ± 2.6	2295.9 ± 658.2
	Lensectomy	35		0.01 ± 0.20	12.6 ± 2.8	2244.1 ± 622.0

N.A. = not applicable; PI = peripheral iridectomy; LI = laser iridotomy; BSCVA = best spectacle-corrected visual acuity; logMAR = logarithm of the minimum angle of resolution.

## Data Availability

The data that support the findings of this study are available from the corresponding author, KK, upon reasonable request.
